# Supplementary material for: Camera Trapping Reveals Spatiotemporal Partitioning Patterns and Conservation Implications for Two Sympatric Pheasant Species in the Qilian Mountains, Northwestern China
Source: Animals (Basel). 2022 Jun 28;12(13):1657. doi: 10.3390/ani12131657 (PMC9264835; doi:10.3390/ani12131657)
Supplement: Supplementary file 1 [file animals-12-01657-s001.zip › animals-1737621-supplementary.pdf]

Supplementary Materials

Table S1. Detailed information on camera traps layout and monitoring throughout the study period.

Study area	Landscape types	Altitude range(m)	Altitude (m) Mean±SD	Number of camera site	Model of infrared camera	Monitoring time range	Camera-days
QF	Scrub-Grassland	2400-3000	2783±201.21	20	Ltl 6210	2017-08-2019-08	6,267
LCH	Scrub-Grassland	2900-3600	3315±168.43	20	EREAGLE E1B	2018-12-2020-08	6,158
SDL	Coniferous forests	2800-3500	3027±176.64	20	Ltl 6210	2017-08-2019-08	11,134
MC	Scrub-Grassland	3000-3800	3375±160.17	40	EREAGLE E1B	2018-11-2019-03	2,300
XYH	Coniferous forests	2500-3300	2800±213.82	17	EREAGLE E1B	2017-11-2019-07	3,601
HX	Coniferous forests	2700-3400	3060±171.50	20	Ltl 6511	2017-08-2019-08	9,746
Total	——	2400-3800	3116±284.61	137	——	2017-08-2020-08	39,206

Table S2. Environment variables used for the MaxENT modeling for EP and BP.

Category	Variable	Units	Symbol	EP	BP	Source
Bioclimatic data	Annual mean temperature	℃	bio1	—	Y	www.worldclim.org/
	Mean diurnal range	℃	bio2	—	—	www.worldclim.org
	Isothermality	℃	bio3	—	—	www.worldclim.org
	Temperature Seasonality	℃	bio4	Y	—	www.worldclim.org
	Max Temperature of Warmest Month	℃	bio5	Y	—	www.worldclim.org/
	Min Temperature of Coldest Month	℃	bio6	—	—	www.worldclim.org
	Annual Temperature Range	℃	bio7	—	—	www.worldclim.org
	Mean Temperature of Wettest Quarter	℃	bio8	—	—	www.worldclim.org
	Mean Temperature of Driest Quarter	℃	bio9	—	—	www.worldclim.org/
	Mean Temperature of Warmest Quarter	℃	bio10	—	—	www.worldclim.org
	Mean Temperature of Coldest Quarter	℃	bio11	—	—	www.worldclim.org
	Annual Precipitation	mm	bio12	—	Y	www.worldclim.org
	Precipitation of Wettest Month	mm	bio13	—	—	www.worldclim.org/
	Precipitation of Driest Month	mm	bio14	—	—	www.worldclim.org
	Precipitation Seasonality	mm	bio15	Y	—	www.worldclim.org
	Precipitation of Wettest Quarter	mm	bio16	—	—	www.worldclim.org
	Precipitation of Driest Quarter	mm	bio17	—	—	www.worldclim.org
	Precipitation of Warmest Quarter	mm	bio18	—	—	www.worldclim.org
	Precipitation of Coldest Quarter	mm	bio19	Y	Y	www.worldclim.org
Vegetation	Global land cover		glc	Y	Y	www.globallandcover.com/
	Normalized difference vegetation index		ndvi	Y	Y	www. resdc.cn/
Topographical data	Altitude above mean sea level	m	altitude	Y	Y	www.gscloud.cn/
	Slope of the terrain	°	slope	Y	Y	Extraction in spatial data
	Cardinal orientation of the slope	°	aspect	Y	Y	Extraction in spatial data
	Distance to rivers	m	dis_riv	Y	Y	Euclidean distance
Human disturbance	Distance to settlements	m	dis_set	Y	Y	Euclidean distance
	Distance to roads	m	dis_roa	Y	Y	Euclidean distance
	Human influence index		hii	Y	Y	sedac.ciesin.columbia.edu/

Note: Y indicates the used variable.

Table S3. List of species, conservation status according to the IUCN, CITES and PCC, and the number of detection sites and records for Galliformes in the QMNNR of Northwestern China from August 2017 to August 2020.

Common name	Scientific name	Conservation status			Numbers of Camera Sites Captured [Detection records]					
		IUCN[1]	CITES[2]	PCC[3]	QF	LCH	SDL	MC	XYH	HX
Blue Eared Pheasant	*Crossoptilon auritum**	LC		II	11 [127]	11 [107]	13 [59]	1 [8]	13 [85]	20 [99]
Blood Pheasant	*Ithaginis cruentus*	LC	II	II	—	—	7 [56]	—	5 [5]	6 [45]
Chinese Grouse	*Tetrastes sewerzowi**	NT		I	—	—	3 [7]	—	2 [7]	4 [20]
Chestnut-throated Partridge	*Tetraophasis obscurus**	LC		I	—	—	4 [15]	—	—	4 [11]
Chukar Partridge	*Alectoris chukar*	LC			4 [7]	—	1 [7]	—	—	—
Himalayan Snowcock	*Tetraogallus himalayensis*	LC		II	5 [10]	—	—	—	—	—
Tibetan Snowcock	*Tetraogallus tibetanus*	LC	I	II	—	1 [1]	1 [1]	—	—	—
Tibetan Partridge	*Perdix hodgsoniae*	LC			—	1 [1]	—	—	—	—

Note: The "*" represents endemic to China.

[1]The IUCN is recorded by the website www.iucnredlist.org/ (VU vulnerable, NT near threatened, LC least concern) (IUCN 2021).

[2]The CITES is recorded by the website of checklist.cites.org/.

[3]The full name of the PCC is the protection class in China (http://www.forestry.gov.cn).

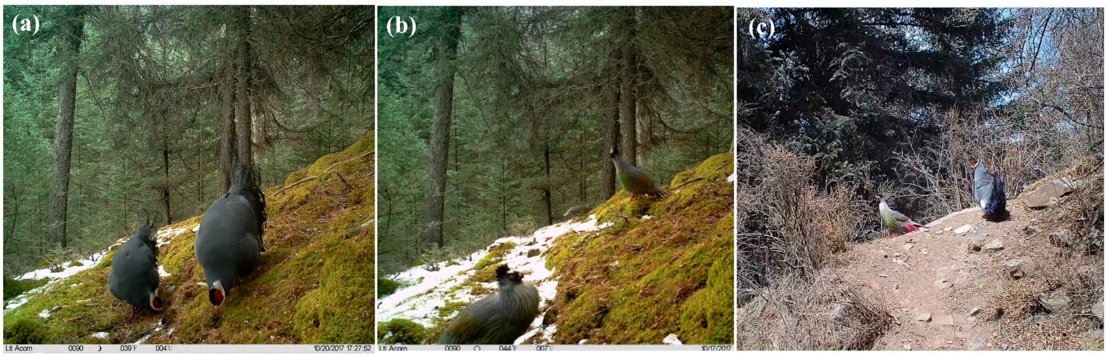

Figure S1. Two sympatric pheasant species captured by camera traps in the QMNNR of Northwestern China. Photographs of (a) EP and (b) BP foraging in the same site during late breeding period. (c) The co-occurrence photograph of both species in the same frame.

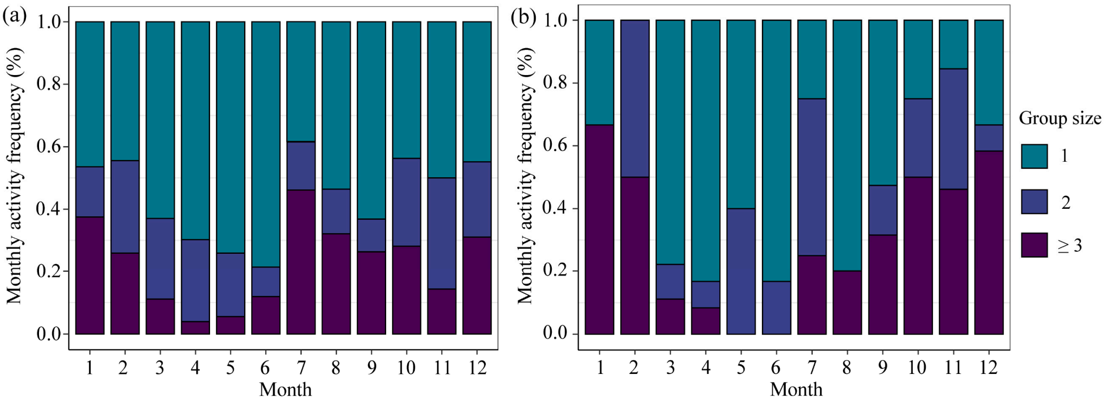

Figure S2. Monthly variation in the number of detected group sizes for (a) EP and (b) BP based on detection records captured by camera traps in the QMNNR of Northwestern China from August 2017 to August 2020.

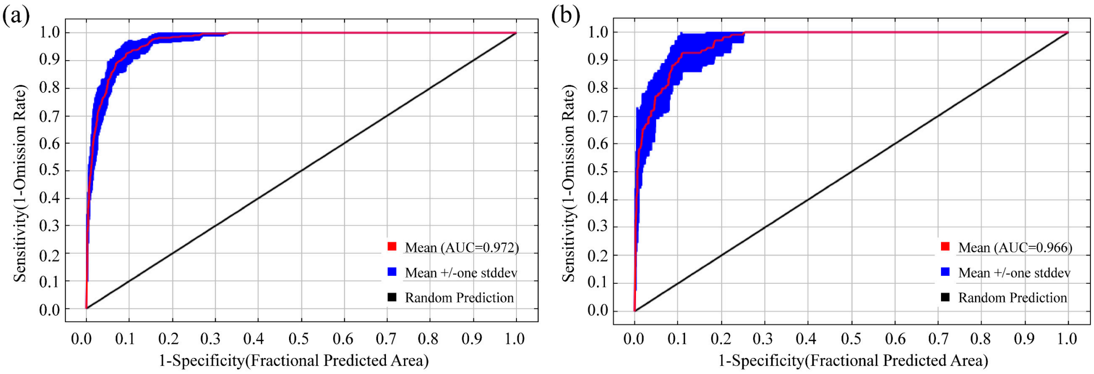

Figure S3. Model performance based on the area under the curve (AUC) for (a) EP and (b) BP.
